# Experiences of using life histories with health workers in post-conflict and crisis settings: methodological reflections

**DOI:** 10.1093/heapol/czw166

**Published:** 2017-01-04

**Authors:** Sophie Witter, Justine Namakula, Alvaro Alonso-Garbayo, Haja Wurie, Sally Theobald, Wilson Mashange, Bandeth Ros, Stephen Buzuzi, Richard Mangwi, Tim Martineau

**Affiliations:** 1 Professor of International Health Financing and Health Systems, ReBUILD and Queen Margaret University, Edinburgh, UK; 2 Research Fellow, ReBUILD and Department of Health Policy, Planning and Management, Makerere School of Public Health, Kampala, Uganda; 3 ReBUILD, Liverpool School of Tropical Medicine, Liverpool, UK; 4 Health Systems Researcher, ReBUILD Consortium and College of Medicine and Allied Health Sciences, University of Sierra Leone, Freetown; 5 Social Science and International Health, ReBUILD and RinGS consortia, Liverpool School of Tropical Medicine, Liverpool, UK; 6 Institute of Development Studies, Sussex, UK; 7 ReBUILD and Biomedical Research and Training Institute, Harare, Zimbabwe; 8 ReBUILD and RinGS consortia, Cambodian Development Resource Institute, Phnom Penh, Cambodia; 9 Public Health Researcher, ReBUILD and RinGS consortia, Biomedical Research and Training Institute, Harare, Zimbabwe; 10 ReBUILD and Department of Health Policy, Planning and Management, Makerere School of Public Health, Kampala, Uganda; 11 Rebuild and Liverpool School of Tropical Medicine, Liverpool, UK

**Keywords:** Health workers, life histories, post-conflict, qualitative methods

## Abstract

**Introduction:** Life history is a research tool which has been used primarily in sociology and anthropology to document experiences of marginalized individuals and communities. It has been less explored in relation to health system research. In this paper, we examine our experience of using life histories to explore health system trajectories coming out of conflict through the eyes of health workers.

**Methods:** Life histories were used in four inter-related projects looking at health worker incentives, the impact of Ebola on health workers, deployment policies, and gender and leadership in the health sector. In total 244 health workers of various cadres were interviewed in Uganda, Sierra Leone, Zimbabwe and Cambodia. The life histories were one element within mixed methods research.

**Results:** We examine the challenges faced and how these were managed. They arose in relation to gaining access, data gathering, and analysing and presenting findings from life histories. Access challenges included lack of familiarity with the method, reluctance to expose very personal information and sentiments, lack of trust in confidentiality, particularly given the traumatized contexts, and, in some cases, cynicism about research and its potential to improve working lives. In relation to data gathering, there was variable willingness to draw lifelines, and some reluctance to broach sensitive topics, particularly in contexts where policy-related issues and legitimacy are commonly still contested. Presentation of lifeline data without compromising confidentiality is also an ethical challenge.

**Conclusion:** We discuss how these challenges were (to a large extent) surmounted and conclude that life histories with health staff can be a very powerful tool, particularly in contexts where routine data sources are absent or weak, and where health workers constitute a marginalized community (as is often the case for mid-level cadres, those serving in remote areas, and staff who have lived through conflict and crisis).

Key Messages

- The life histories provided a rich source of information, capturing health sector histories through the personal perceptions of the staff working in them. As a tool for research on changes over a period of time and in data-scarce contexts, they were a key component within our mixed methods research.
- The vivid experiences which participants have undergone provide very moving evidence – evidence which may be known locally, but by being documented can dignify those who lived through them.
- Although health workers are not normally be assumed to be a vulnerable group whose voices are unheard, mid-level cadres in remote areas are commonly marginalized from the centres of power in their countries. Often targeted during times of conflict, unrecognized for staying in post afterwards, unsupported and without effective communication and supervision, they feel forgotten.
- The life histories build on one of the most essential human characteristics – telling stories and making sense of the world through our own life experiences. They have shown their potential to contribute to health system research.

## Introduction

Life history is a qualitative method used often in sociology and anthropology. A life history is a case study based on the story of an individual, hence they focus on individuality, subjectivity and the particular experience (Plummer, 1983). It uses a narrative research approach where interviewees narrate their own history, sometimes also using visual aids such as the drawing of a lifeline to illustrate major events experienced. Previously, life histories have been used as part of a mixed methods approach in chronic poverty research in Uganda, Zimbabwe and Bangalore, particularly in relation to poverty trajectories of households over time–vulnerability and resilience in relation to shocks (Bird, 2008, Kessy and Tarmo, 2011, Benjamin, 2003) as well as assets, gender and poverty (Doss *et al.* 2011). There is also increasing interest in life histories from researchers influenced by feminist epistemologies - to address the ways in which power and positionality shape research encounters and to capture experiences and perceptions that are often unheard (Ssali and Theobald, 2016).

In health research, life histories have been used in areas such as mental health (Chafetz, 1996, Solsulki, Buchanan and Donnell, 2010, (in)fertility (Hemmings, 2007) and in exploring socio-economic determinants of health. However, they have not been widely used in health systems research, which we understand as research "concerned with how health services are financed, delivered and organized and how these functions are linked within an overall health system with its associated policies and institutions" (Mills *et al.* 2008).

When the focus of the interview is the subject’s career or professional trajectory and main work-related events they are referred to as "work histories". Work histories have been used in multiple disciplines from hospitality to industrial relations. In the health sector work histories have been used most often in occupational and environmental health (e.g. also called "occupational histories") (Harris *et al.* 2016, Martin *et al.* 2012), often using surveys. There is very limited literature about the use of work histories in health workforce research. Three studies were found that used mixed methods including job (also sometimes called career or work) histories using qualitative methods in India, two of which are published (Kadam *et al.* 2016, Purohit and Martineau, 2016).

In the ReBUILD programme^1^, we wanted to investigate how health systems had been rebuilt post-conflict through the lenses of experiences of both households and health workers, including their gendered experiences. The life history approach appealed in allowing us to tap into the experiences of health workers who had experienced conflict and post-conflict periods of their country’s history and could explain how they had been affected, both by the conflict and the human resources for health (HRH) policies introduced in its aftermath. The fact that routine data systems were weak and disrupted in these health systems made the life histories even more valuable. Life histories were also thought to be conducive to gender analysis as participants are enabled to narrate in their own voices their experiences of work (and war or fragility) and how gender shaped their experiences (Ssali and Theobald 2016).

Life histories are placed within the sociocultural theory framework which seeks to understand how human actions are related to the social context in which they take place (Moen, 2006). From an epistemological perspective, life history is an interpretive auto-ethnographic method (Denzin, 2014) which has the potential to connect the stories of individuals to social phenomena, linking the micro and the macro. Within a social constructivist paradigm (Vygotsky, 1978), the researcher makes sense of the stories narrated by participants on their experience of how they interacted with the different social structures and historical processes within which they were situated. These stories, articulated in the present, constitute the life histories (Green and Thorogood, 2009).

In our case, the narrative of the interaction of the health workers with the social environment in which they lived and worked through their professional life allowed us to understand how they experienced the process of post-conflict reconstruction. In addition, through their lives and experiences we also sought to obtain an understanding of the evolution of the health system and the different processes related to the work environment. Their lived experiences provided us with a personal perspective on the effectiveness and intended as well as the (un) intended consequences of human resource policies and their evolution.

This paper presents some of the challenges and lessons learned in the process of using the life history approach with health staff in Sierra Leone, Zimbabwe, northern Uganda and Cambodia.

## Methods

Four countries were selected to represent different contexts and stages of distance from conflict within the ReBUILD research programme: Zimbabwe, which experienced economic and political crises, peaking in 2008; northern Uganda, which emerged from the Lord’s Resistance Army-sponsored war in 2006; Sierra Leone, whose civil war ended in 2002 and was recently affected by an Ebola outbreak in 2014 - 2015; and Cambodia, where a comprehensive peace post-Khmer Rouge began in 1999.

Five studies were carried out by ReBUILD using life histories as a method (Table 1). A mixed methods study on health worker incentives was designed, using both retrospective and cross-sectional tools, one of which was life histories with health workers in four countries. The objective of the overall research was to understand changing health worker incentives and their policy implications in the post-conflict and post-crisis period (Witter *et al.* 2012). This was extended by research into the impact of Ebola on health workers in Sierra Leone. Following this, two additional qualitative studies (both using the life history approach) and supported through the RinGs network were undertaken with gender as a primary focus: one on gender, human resources for health and leadership in Cambodia (Vong and Ros, 2016), and a second on gender and human resources for health in rural Zimbabwe (Buzuzi *et al.* 2016). A fifth project was carried out in Zimbabwe and Uganda to understand changing deployment policies and their implications in the post-conflict and post-crisis period (Martineau *et al.* 2012).

**Table 1.** Summary of life histories used in the ReBUILD and RinGS studies

	Cambodia	Sierra Leone	Uganda	Zimbabwe
**Site selection**	Six provinces (covering all four ecological regions) – one district from each, including urban, rural and those with more or less external support. The RinGS project was in one province, covering 2 operational districts.	Four districts (covering all main regions, including urban vs rural/hard to reach and areas of varied socioeconomic status)	Three districts in Acholi sub-region – most conflict-affected area	Two provinces – one well served and one under-served; three districts including urban, mixed and rural. The RinGs study was done in 4 districts in the Midlands Province. Rural deployment study was done in three districts in Midlands province
**Sectors included**	Public sector only	Public sector only	Public sector and private not-for-profit	Public (government, municipal and rural district council employees), mission and private sector
**Timeframe**	1979 onwards	2000 onwards (last phase of conflict; post-conflict since 2002)	2000 onwards (six years during; six years after conflict)	1997 onward (economic crisis, and post - since 2009)
**Health workers interviewed **Total: 253	HWI: 19 (doctors, medical assistants, nurses, midwives) RinGS project: 20 Deployment: 20 (same cadres as HWI) Total: 59	HWI and Ebola follow on: 48 (doctors, nurses, midwives, community health officers – CHOs) Total: 39	HWI: 26(clinical officers, nurses, nursing assistants, midwives and others) Total: 26	HWI: 34 (doctor, nurses, midwifes environmental health practitioners and clinical officers) RinGs study: 19 Rural deployment: 67 (nurses, midwives, EHPs, PCNs) Total: 120
**Health worker incentive project**: Life histories were deployed to explore health workers’ perceptions and experiences of their working environment, how it has evolved and factors which would encourage or discourage them from staying in post in remote areas and being productive. They were encouraged to produce visual aids, such as timelines. These were conducted with health workers meeting specific criteria (including length of service in the area, to capture experiences of conflict and post-conflict periods,) in selected health care facilities in the study areas using an open-ended topic guide. The topic guide covered the following areas:				
How they became health workers Their career path since, and what influenced it, including the role of gender What motivates/discourages them to work in rural areas and across different sectors The challenges they face in their job and how they cope with them Conflict related challenges and how they coped Their career aspirations Their knowledge and perceptions of recent and current incentives.				
**Impact of Ebola**: An extension project was carried out in Sierra Leone during the Ebola outbreak to understand the challenges to a responsive and resilient health system from a health worker perspective and how to build resilience to similar shocks in future. For the Ebola follow-on study the following topics were covered with staff working in Ebola treatment or isolation centres:				
Impact of Ebola on communities and health workers and impact of existing challenges on the response Strategies adopted by health workers to cope with these effects Recommendations for the rebuilding of the health system, focusing on building trust with communities and strengthening the health workforce				
**Gender and career development**: The life histories in the RinGs projects in Zimbabwe and Cambodia included a specific gender lens, with the focus in Cambodia on gender and leadership and in Zimbabwe on relocation, rural deployment and promotion. Both studies included specific probes and discussion on issues relating to the implementation of equal opportunities policies and legislation and how gender roles and relations, expectations and roles at (overlapping) household, organizational and socio-cultural levels affect health workers’ access to training, promotion and career advancement opportunities.				
**Deployment:** The project on deployment of health workers in Zimbabwe and Uganda took a similar approach to the study on incentives, selecting where possible health workers who could discuss the different jobs they had had during their careers, during and after the conflict/crisis periods, and how they moved or were transferred by the authorities between these jobs. Health workers were selected from government facilities and those run by faith-based organizations. In the study in Zimbabwe the health workers were encouraged to draw a timeline which included the various jobs, training courses and promotions. The analysis looked at how the conflict and the crisis respectively had affected policy and systems development and implementation, whether deployment rules had been followed, and what were the challenges and opportunities that these specific settings provided.				

Ethical approval was gained from each country ethics board and the relevant UK universities. The interviews were tape recorded and noted after gaining permission from the participants. The interviews took place in a private place acceptable to the interviewee, such as their office. Thematic analysis using manual coding, NVIVO and ATLAS Ti in Uganda was carried out on transcribed (and sometimes translated) texts. The analysis started from the themes of the topic guide, but developed in each case to reflect the topics raised in the interviews.

This paper was drawn from the experiences of ten local and international researchers who had worked on the life histories in these post-conflict settings. Ethical and practical research issues were shared during annual workshops over the past three years. This culminated in a round-table discussion in June 2016 in which challenges and solutions were shared, structured according to the research process stages.

## Reflections on challenges and lessons

The experience of using life histories in these contexts threw up ethical and practical issues about gaining access to and trust of participants, about the management of the data gathering process, and also how to analyse and present findings appropriately from life histories.

### Gaining access/willingness to participate

The life histories are by necessity very personal accounts which mix personal, family and professional events and experiences. The normal process of establishing trust and confidence in the researcher was therefore of great importance, especially as the topics covered some sensitive and traumatic issues, such as experiences in wartime and HIV and AIDS infection. Moreover, in these settings, where there are few staff, individuals are relatively easy to identify. Some staff were unwilling to participate and others were willing to participate but not to be recorded.

In other cases, there was a simple lack of familiarity with the method, which caused initial hesitation, or a misunderstanding of purpose. If staff had participated in research at all, it was mostly in the form of surveys, so this in-depth qualitative approach does require further explanation to enable participants to feel at ease, address concerns and support trustworthy interactions. In Cambodia, for example, men were initially reluctant to be interviewed for the gender study, assuming that this should focus only on women, and that interviews with male mangers would be accusatory. There was therefore always an explanation to the participants in the beginning about what we mean by the concept of gender to enable shared understanding and research processes that build trust and enable open discussion on how gender roles and relations shape experiences; The importance of including both male and female perspectives and life stories was also stressed. Assurances of confidentiality have to be more explicit than usual as the information given is very personal.

In some contexts, like Zimbabwe, we experienced a climate of fear in relation to speaking out by health workers, particularly in revealing negative experiences about public policies. A typical area that participants were unwilling to discuss was that related to security, which is nevertheless integral to talking about conflict/crisis and post-crisis experiences, and also the causes of the crisis (Chirwa *et al.* 2015). Managers also blocked access to staff in some cases, especially in the mission and private sector in Zimbabwe (Chirwa *et al.* 2015). In Cambodia, concern was expressed by managers about labour unrest.

Staffing rosters have of course to be respected. As staff are overloaded in these settings, it may be necessary to be particularly flexible on timing, especially for senior staff. To conduct the interviews, the research teams made appointments over the weekend, when health workers were ‘off duty’ and at their homes. This adds time to data collection but may considerably improve participation and the quality of the evidence as participants felt freer to discuss their experiences away from the formal workplace (Namakula and Witter, 2014, Namakula and Witter, 2014). More than one appointment can be made, if needed, however, this is not ideal if the flow of recollection is interrupted. We also had to be flexible in our approach to recording – taking notes, where necessary, rather than having a full transcript, if this made the participant feel more relaxed and willing to talk.

As in other settings, hierarchy has to be observed in gaining access – providing letters of introduction and official agreement for the study to proceed. The positionality of the researcher is also critical; and we thought carefully about the age and gender of the interviewer. In these contexts, other aspects of positionality also matter: for example, being a diaspora Sierra Leonean was unhelpful in some cases as the staff resented the fact that their interviewer had escaped the challenges and trauma of the conflict and post-conflict years.

There was also considerable cynicism in the Zimbabwean context, and to a lesser extent in the other three, about the extent to which the research would feed back into practice and benefit the participants. This may be linked to ‘research fatigue’ but also to disillusionment with the policy processes and implementation (there is a lack of confidence in public capacity to implement and sustain reforms, particularly in these settings, where trust has been eroded and financial constraints are often severe). The participants in some cases felt that they are often asked about their challenges, but nothing is done to alleviate them.

Convincing staff in these environments was more challenging for senior staff, who may be not only cynical but also overloaded and very busy (the life histories are intense, as discussed below).

Cynicism is hardest to address as the researcher is in no position to be able to guarantee research impact; in these settings it is best to be clear about how research will be used and fed back to the participants but to acknowledge the actions which lie outside the researchers’ control. It may also mean focusing on research methods which are more closely tied to policy issues, such as operational research, and giving increased time and resources to research uptake, and to reporting back to communities to close the research loop. In some cases, however, we may have to accept that some profiles of staff will not participate, which may have implications for the kinds of insights gathered and needs critical reflection in the analysis process.

### Data gathering: getting the full picture

Most, but not all, of the life history interviews involved participants being invited to draw a lifeline with key events as they had experienced them. These lifelines served as a prompt and talking point during the interview, as well as a visual record which could be analysed afterwards.

Most participants were willing to draw but others lacked confidence or preferred the researcher to put down events as directed by the interviewee. In Uganda, for example (Namakula *et al.* 2013), the majority of participants were reluctant to draw the lifelines. This is because they feared that ‘they would do it wrong’ and that they knew that ‘the researchers would do it better’. They requested the interviewers to draw the life lines while they did the ‘story telling’. A rough lifeline was drawn during the interview to guide the discussion and later made neater. In some contexts, for example Zimbabwe, men showed more confidence in drawing than women.

The life history can be intensive in time – most took 1-3 hours, which can be challenging for staff. Whether the lifelines added to the time or made it shorter was not clear as no comparison with other methods was done. However, the lifelines probably improved the quality of the data collected. They created a visual and interactive activity which supported further detailed discussion. Participants were triggered to think of the episodes they had missed – to go back and fill in details, and discuss the reasons behind choices and changes. Lifelines were used to trigger recall during the interview as participants looked at the lines and made adjustments to information, which probably made more efficient use of the interview time. The lifelines also guided the interviewer in probing (as information gaps were clearly visible) and enabled the interviewer to check for consistency.

The method of using life history and timelines was new to participants. Those who engaged, and especially the less senior staff, such as in Uganda, seemed eager to be involved and to find out what happened at the end (Namakula *et al.* 2013). It was a method that enabled them ‘tell their story’. At the end of the interviews, participants were given the complete timeline and many expressed surprise at how much they had experienced in their life, how much they had moved between jobs etc. Other researchers have highlighted the therapeutic benefits of life histories that enable participants to identify, discuss and locate social meaning to events (Ssali and Theobald, 2016, Goodson and Gill, 2011). Many participants appreciated the process and the opening and reflection process enabled by life histories, although there were some key ethical issues, particularly in relation to asking staff to recall traumatic situations. In Sierra Leone, for example, health workers were asked to discuss the impact of Ebola, which was recent and visibly distressing (Wurie, Witter and Raven, 2016). In Uganda, participants recalled traumatic experiences of conflict such as abduction, near death, ambushes and loss of colleagues and family members (Namakula and Witter, 2014)). In many instances, participants paused for minutes and then continued. Developing trusting and empathetic relationships is critical; as is thinking critically about positionality. Interviewers were trained to establish an empathetic relationship during the interview and also asked the participant if they were comfortable continuing or pausing for some time. There was no formal counselling available for respondents or the study teams, however teams had access to wider support and debriefing to discuss traumatic experiences. Enabling access to assistance for participants, where needed, would be appropriate in future.

The longest period of recall was expected to be problematic – participants were being asked to talk about events which had occurred in some cases many decades ago. However, as the events are all selected as being of high personal and emotional significance, they remained vivid. The objective of the tool is in any case focused on gathering personal impressions from the perspective of the individuals, i.e. supporting internal (depth and detail of experience), rather than external validity.

In some still very sensitive environments, like Zimbabwe, interviewees would choose not to give too much detail about the negative issues related to policy implementation and practices, or they would give details but indicate that they were ‘off the record’ or ask the interviewer to stop recording. In these cases, information was noted but not used in reports, which can be frustrating as these exchanges provide very material insights into issues of importance to participants.

### Data analysis and presentation of results

During data analysis, interview transcripts were analysed for converging and diverging themes across individuals with different characteristics. The use of the lifelines was more holistic – they retain the overview of each person’s life and therefore offer a more integrated perspective. The lifelines provide a chronological overview, which can easily be read for associations (e.g. improved motivation after implementation of a certain policy, or how gender roles and relations shape experience of promotion). They help to tell the story from beginning to the end. In some cases, information from the lifelines was entered into Excel – for example, in the deployment study, this was done to give information on transfers. For the health worker incentive study, the number of changes of post per person over time, or location of initial training, were analysed from the lifelines.

Presenting the lifelines in reports was however problematic as the information which they contain is very revealing. To omit the details is to lose their value; to include them makes the individual all too identifiable. For that reason, while the lifelines were used as aids in the interviews and for analysis, they were not included in the final reports and articles. Smaller numbers of health staff in many post conflict contexts mean that they may be easily identifiable and in many cases in the writing up process less detail was provided about the participant, to ensure that confidentiality was maintained and potential harm to the participants avoided.

## Discussion and Conclusion

Overall the life histories provided a very rich source of information, capturing health sector histories through the personal perceptions of the staff working in them. As a tool for research on changes over a period of time and in data-scarce contexts it was invaluable; similar positive experiences were documented working with households in northern Uganda (Ssali and Theobald, 2016).

It is important to note however that the life histories were just one of the research tools used to answer the overall research questions, and were combined with other sources with complementary perspectives and strengths (for example, in the health worker incentive project a health worker survey was also carried out, alongside analysis of routine HRH data, key informant interviews, stakeholder mapping and document reviews). It was therefore part of a mixed methods study (Witter *et al.* 2012). Similarly, in the deployment study data on transfer frequencies was also derived from a survey of personnel records (Martineau *et al.* 2012), while the RInGS case studies also used key informant interviews and document review.

Many of the challenges noted above would have been encountered in any in-depth qualitative method (and indeed reflect many of the ethical issues raised in Molyneux *et al.* 2016) and the solutions are also of wider applicability. In-depth qualitative research is always intensive, and the trustworthiness of the approach is tied directly to the skills and trustworthiness of the researcher and hence requires considerable investment in time, selecting researchers with the necessary qualities and also thorough training (Patton, 1990). There is also need to think critically about positionality and the role of power relationships, gender and age in the research encounter, as well as the actual setting of the life history to ensure trusting and empathetic relationships are established (Ssali and Theobald, 2016). Some of the challenges of gaining access which we encountered relate more specifically to the mind-set of health staff, especially at senior levels. They are more likely to be busy, research-fatigued and (perhaps) cynical.

The extent to which the challenges noted are specific to post-conflict or conflict-affected settings is debatable. Certainly some of these contexts present a difficult environment for research in that underlying social divisions may not be healed or may have been papered over. In this case, there is little confidence that research evidence can play a significant role in changing policy and practice, in the face of chronic political, social and economic bottlenecks.

At the same time, the vivid experiences which participants have undergone provide very moving evidence – evidence which may be known locally, but by being documented not only can move minds but also dignify those who lived through them. Although health workers may not normally be assumed to be a vulnerable group whose voices are unheard, mid-level cadres in remote areas are marginalized from the centres of power in their countries – something which our reports make very clear (Namakula *et al.* 2013, Wurie and Witter, 2014, Chirwa *et al.* 2015, So and Witter, 2016). Often targeted during times of conflict, unrecognized for staying in post afterwards, unsupported and without effective communication and supervision, they commonly feel forgotten and have experienced intense vulnerabilities in different ways.

The life histories build on one of the most essential human characteristics – telling stories and making sense of the world through our own life experiences. They have shown their potential to contribute to health system research.

## Notes

1
www.rebuildconsortium.com
